# Do Pregnant Women and Those at Risk of Developing Post-Natal Depression Consume Lower Amounts of Long Chain Omega-3 Polyunsaturated Fatty Acids?

**DOI:** 10.3390/nu2020198

**Published:** 2010-02-21

**Authors:** Victoria F. Cosatto, Paul L. Else, Barbara J. Meyer

**Affiliations:** 1 Now at Centre for Vision Research, Westmead Millennium Institute, University of Sydney Westmead Hospital, Hawkesbury Rd, Westmead NSW 2145; Australia; Email: victoria_cosatto@wmi.usyd.edu.au; 2 School of Health Sciences and Metabolic Research Centre, University of Wollongong, NSW 2522; Australia; Email: paul_else@uow.edu.au

**Keywords:** long chain omega-3 polyunsaturated fatty acids (LC n-3 PUFA), pregnant women, postnatal depression

## Abstract

The aims were to compare intakes of long chain omega-3 polyunsaturated fatty acid (LC n-3 PUFA) in pregnant and non-pregnant women in Australia and to compare these intakes to the Australian National Nutrition Survey of 1995 (NNS95) [1] and to determine if the LC n-3 PUFA intakes differed in women who may be ‘at risk’ compared with women ‘not at risk’ of developing post-natal depression (PND). A validated LC n-3 PUFA food frequency questionnaire and pregnant women’s Edinburgh Postnatal Depression Scale (EPDS) scores were used. LC n-3 PUFA intakes were comparable to the NNS95 but did not differ due to pregnancy or whether or not a woman is at risk of developing PND.

## 1. Introduction

The health benefits of consuming long chain omega-3 polyunsaturated fatty acids (LC n-3 PUFA), particularly eicosapentaenoic acid (EPA, 20:5n-3) and docosahexaenoic acid (DHA, 22:6n-3), are numerous and include cardiovascular, immune and putative mental health benefits [2,3,4,5,6,7] EPA and DHA can reduce the risk of coronary heart disease by up to 25% and sudden cardiac death by approximately 45% [8,9].

LC n-3 PUFA are also essential for proper growth and development of the foetus. During pregnancy the circulating levels of maternal EPA and DHA are upregulated [10,11] and selectively transferred to the developing foetus via the placenta [12] to be incorporated into tissues such as the photoreceptors of the retina [13] and membranes of the central nervous system [14]. LC n-3 PUFA supplementation studies during pregnancy have shown prolonged gestation [15], increased birth weight and length [15] and improved visual acuity of the newborn [16].

Both EPA and DHA can be desaturated and elongated from shorter chain n-3 PUFA precursor known as α-linolenic acid (ALA, 18:3n-3) [17,18,19], although the physiological relevance of this conversion is questionable. Studies by Burdge *et al.* [17,18,19] show that in men less than 6% of ALA is converted to EPA and only 0.05% converted to DHA, although in women the conversion rate is much higher at 21% and 9% respectively. Therefore to obtain the potential health benefits from LC n-3 PUFA, consumption of preformed EPA and DHA is better than relying on the conversion of ALA to LC n-3 PUFA.

During pregnancy, women may need to supplement their diet with extra LC n-3 PUFA in order to cater for the extra demand of the foetus in addition to their own physiological needs [10]. Fish and seafood is the best source of the LC n-3 PUFA, followed by meat and eggs [20]. A recent study by Hibbeln *et al.* [21] showed that women who consumed more than 340g of fish/seafood per week (equivalent to two fish meals a week) during their pregnancy, had offspring with higher IQ at age 8 compared with the offspring whose mothers consumed less than 340g per week of fish/seafood.

Observational studies have shown that LC n-3 PUFA serum and phospholipid levels are lower in people who suffer from depression compared to healthy control subjects [22]. It is possible that during pregnancy women with lower tissue levels of LC n-3 PUFA are more at risk of developing postnatal depression (PND). Certainly DHA is mobilised in the maternal circulation during pregnancy [12] and if DHA is being released from the maternal brain to meet the demand of the foetus this could contribute to the development of PND, but more research is warranted. Investigators believe that supplementing the diet with LC n-3 PUFA enriched foods may prevent this [23].

The consensus statement for dietary fat intakes for pregnant and lactating women suggests an intake of DHA of at least 200mg/day [24]. Recently in Australia there has been some acknowledgement of the health benefits of the consumption of DHA and other LC n-3 PUFA during pregnancy [25], yet documented evidence regarding pregnant women and their consumption of LC n-3 PUFA is lacking. Currently, there does not appear to be a link between LC n-3 PUFA intakes and postnatal depression [26], however this is based on a very limited number of studies. 

Therefore the aims of this study were (1) to determine the intakes of LC n-3 PUFA in pregnant and non-pregnant women in Australia; (2) to compare these intakes to those of Australian females assessed in the Australian National Nutrition Survey of 1995 (NNS95) [1] and (3) to determine if the LC n-3 PUFA intakes differed in women diagnosed ‘at risk’ of developing PND compared with women ‘not at risk’ of PND.

## 2. Methods

### 2.1. Subject Recruitment

Pregnant women were recruited from the South-West Sydney Area Health Service in Australia whilst attending their first antenatal doctor’s visit at the outpatients’ clinic. All women booked in for their initial doctor’s visit were given an information sheet, by antenatal clinic reception staff, outlining the background to the study. A researcher then approached the women to ask if they would like to participate in the study by completing a food frequency questionnaire (FFQ). 

Non-pregnant women were recruited through both an e-mail and poster advertisement campaign both at the University of Wollongong and local community. Subjects were required to have maintained a constant diet over the previous 3 months; apart from this there were no other exclusion criteria. Ethics approval for this study was granted both from the University of Wollongong and South-West Sydney Area Health Service.

### 2.2. Administration of the Demographic and LC n-3 PUFA Food Frequency Questionnaire (FFQ) to Pregnant and Non-Pregnant Women

LC n-3 PUFA intakes in women were determined using a newly validated FFQ [27,28]. Volunteers were asked to complete the tailored FFQ during their antenatal clinic visit, or during arranged visits to the University of Wollongong. Other information supplied by the women included age (years), height (cm), weight (kg) and for pregnant women gestation stage (weeks). Digital scales and a wall-mounted stadiometer were used by a researcher to measure weights and heights, respectively, for the non-pregnant women. However, equipment to measure weight and height was not available at the antenatal clinic on the day so measurements were self-reported by the pregnant women. 

### 2.3. Analysis of Daily LC n-3 PUFA Intakes

The Foodworks Fatty Acid Database [29] was used to analyse each subject’s FFQ. To determine the daily consumption rate of each type of food (in grams per day), from the previous 3 months, the portion amount (in grams) was multiplied and/or divided by the frequency of each type of food consumed. The daily consumption intake for EPA, docosapentaenoic acid (DPA, 22:5n-3), DHA and total LC n-3 PUFA were then calculated by multiplying individual fatty acid content, per 100g of food, by the daily food intake per gram. The amount of LC n-3 PUFA was then further divided by 1000 to ascertain the fatty acid content in mg/day.

### 2.4. The Australian National Nutrition Survey 1995 (NNS95)

The Australian NNS95 [30] measured the energy and nutrition intakes of all food and beverages reported by 13, 858 men and women (19 years and over) using a 24-hour recall method. For validation reasons, a sub-sample of participates (n = 8,321) also completed a FFQ reporting consumption for over 100 different food types, as well as vitamins and supplements, proceeding 12 months prior to the study [30]. Howe *et al.* [1] later re-assessed the LC n-3 PUFA content from foods recorded by subjects in both NNS95 24-hour recall and FFQ using a more accurate fatty acid compositional database [1]. Previously, the database used to analysis the NNS95 did not have the fatty acid content for certain foods sources such as meat products [1]. For this reason the LC n-3 intakes of the pregnant and non-pregnant women in this study will be compared to the re-assessed LC n-3 PUFA intakes by Howe *et al*. [1].

### 2.5. The Edinburgh Postnatal Depression Scale (EPDS)

The EPDS is a simple 10 item self-report scale specifically developed to detect the severity and extent of depression during the postnatal period [31]. The EPDS has also been validated for use during the antenatal period [32], as women diagnosed with postnatal depression may have already been depressed during pregnancy [33]. Although the EPDS is not intended to substitute for a full psychiatric assessment, it is used as a tool to assist primary health care workers, without psychiatric experience, to define a population, which needs further evaluation [34]. For this study all pregnant women at the hospital first attend the antenatal clinic to record their medical history and to complete the EPDS. To identify all possible cases of depression, during the antenatal period, the hospital selected a cut-off score of ≥10. Therefore, women who scored 10 or greater on the EPDS were regarded as ‘at risk’ of developing postnatal depression and were immediately referred for counselling. Women, who scored nine or less, on the EPDS, were considered as ‘not at risk’ of developing PND**.** At the time of subject recruitment the researcher was blind to the patient’s EPDS score, so the patient’s medical record number (MRN) was recorded, to assist a second researcher in obtaining EPDS score and/or identifying the number of subjects recruited in each group. The study was unblinded after the FFQs were analysed for the LC n-3 PUFA intakes.

### 2.6. Statistical Analysis

A power calculation using the mean difference of 200mg DHA and a power of 80% at a significance level of 0.05 was completed and 75 women from each group were deemed to be adequate for the study. The data for subject characteristics is presented as mean and standard deviation (SD). The data for EPA, DPA, DHA and total LC n-3 PUFA intakes are presented as mean and standard error of the mean (SEM) as well and the median and the range. Since the data distribution for EPA, DPA, DHA and total LC n-3 PUFA was not normally distributed a Wilcoxon nonparametric test was used to compare the fatty acid data between the pregnant group and the non-pregnant group as well as the LC n-3 PUFA intakes in women ‘at risk’ (EPDS score ≥ 10) and ‘not at risk’ (EPDS score < 10) of developing post-natal depression. All p values < 0.05 were considered significantly different. All statistical analysis was performed using JMP statistical software package version 4.0 (SAS institute, Cary, NC, USA).

## 3. Results and Discussion

### 3.1. Study Participants

During subject recruitment, 133 pregnant women were approached and asked to participate in the study by completing the questionnaires and 115 (86%) agreed to participate. A further, 21 subjects were excluded from the study because they either did not fully complete their questionnaire (n 15) or they did not return the questionnaire (n 6). The total number of women who completed the FFQ and hence were eligible for the study was 94 (82%). For the non-pregnant women, 33 volunteers were recruited for this study. 

The characteristics of the study participants showed no significant differences in age and height in the pregnant and non-pregnant women, but the pregnant women obviously weighed heavier than the non-pregnant women. Characteristics of the study participants are shown in Table 1.

**Table 1:** Characteristics of pregnant and non-pregnant women study participants **^1,2^**.

		Pregnant women			Non-Pregnant Women (n 33)		
Characteristics	(n)	Mean	± SD	Range	Mean	± SD	Range
Age (years)	94	28	± 5	18-41	33	± 11	21–55
Height (cm)	78	164	± 8	146-180	162	± 8	145–176
Weight (kg)	86	79	± 20	45-144	61	± 11	43–90
Body mass index (kg/m**^2^**)	75	29	± 7	20-54	23	± 3	19–31
Pregnancy Stage (weeks)	94	20	± 5	10-34	N/A **^3^**		

**^1 ^**Values are mean ± SD **^2 ^**Note from the pregnant women, 16 women did not report their height, 8 did not report their weight. **^3 ^**N/A Not applicable

### 3.2. LC n-3 Intakes for Pregnant Women, and Non- Pregnant Women and Women from the NNS95

The comparison of the LC n-3 PUFA intakes for the pregnant women, non- pregnant women and women aged 19 years and over from the Australian NNS95 [1] was inclusive of all meat, fish and egg products but excluded all enriched foods and fish oil supplements, as such foods were not available in the NNS95. The mean and median intakes of LC n-3 PUFA (mg/day) consumed by the pregnant women group, are comparable to the average intakes of women aged 19 years and over in the NNS95. The average EPA, DHA and total LC n-3 PUFA intake of the pregnant compared to the non-pregnant women was not significantly different. However, pregnant women’s intakes of DPA were higher (P < 0.05) than the non-pregnant women. A multiple regression analysis did not show any variables such as age, height or weight influencing the DPA intake of the non-pregnant group. This difference in DPA intake could be explained by the amount of meat and seafood products consumed by each group. On average the pregnant women consumed more meat products per day than the non-pregnant women and women from the NNS (174 grams versus 109 grams versus 158 grams, respectively). However, the pregnant women consumed less fish/seafood products per day than the non-pregnant women (35 grams versus 51 grams) and slightly more than the women from the NNS (35 grams versus 26 grams). The comparisons of the mean, median and range of intakes (mg/day) of LC n-3 PUFA for the pregnant women, non-pregnant women and women aged 19 years and over from the Australian NNS95 [1] is shown in Table 2.

**Table 2:** Comparison of LC n-3 PUFA intakes (mg/day) without the inclusion of enriched foods and fish oil supplements for pregnant women with non-pregnant women and the NNS 95 (Howe *et al.*, 2006) **^1^**.

	Pregnant women (n 94)				Non-pregnant women (n 33)				NNS 95 (n 5770)	
Fatty Acid	Mean	SEM	Median	Range	Mean	SEM	Median	Range	Mean	SEM
EPA mg/day	79	6	72	4–321	69	10	53	0–294	60	2
DPA mg/day	85^*^	6	75	3–422	49^*^	7	35	0–146	52	1
DHA mg/day	99	9	75	6–579	124	21	106	0–673	83	3
LC n-3 PUFA mg/day	263	19	235	16-1080	241	36	189	0–1110	195	5

**^1 ^**Abbreviations: LC n-3 PUFA - Long Chain Omega-3 Polyunsaturated Fatty Acid; NNS 95 - National Nutrition Survey 1995 [1]; SEM–Standard Error of the Mean; EPA - Eicosapentaenoic acid; DPA–docosapentaenoic acid; DHA–docosahexaenoic acid; LC n-3 PUFA–sum of EPA+DPA+DHA ^*^ P < 0.05 comparison of pregnant and non-pregnant women (Wilcoxon non-parametric test)

### 3.3. Contribution of LC n-3 PUFA Foods

The relative contribution of LC n-3 PUFA intakes was mainly from foods i.e., meat, fish and eggs, followed by enriched foods and the least amount from fish oil supplements in pregnant women. The main contributor was the natural food sources followed by enriched foods consumed by 48% (n = 45) of the women. Enriched food supplements (Blackmores Ltd. (20 Jubilee Ave, Warriewood, NSW 2102, Australia) pregnancy and lactation formula) were consumed by 5% (n = 5) of the women. The consumption of enriched foods and supplements by these pregnant women provided an additional 8, 1, 29 and 33 mg/day of EPA, DPA, DHA and total LC n-3 PUFA, respectively, to the daily intake (comparison of intakes between Table 2 and Table 3). The pregnant women all consumed at least some type of meat product (100%; n 94), most consumed some type of seafood (88%; n 83) and most consumed eggs (72%; n 68) Table 3.

**Table 3:** Contribution of enriched foods and fish oil supplements in pregnant women to the total LC n-3 PUFA intakes (mg/day) from all sources. **^1^**

Fatty Acid for food group	Mean	SEM	Median	Range
**Meat, fish and eggs (n 94)**				
EPA	79	6	72	4–321
DPA	85	6	75	3–422
DHA	99	9	75	6–579
Total LC n-3 PUFA	263	19	235	16–1080
**Enriched Foods (n 45)**				
EPA	11	2	6	0.22–40
DPA	3	0.5	1	0–16
DHA	38	5	27	0.9–181
Total LC n-3 PUFA	51	7	33	1–234
**Fish oil supplements (n 5)**				
EPA	54	10	64	16–64
DPA	0	0	0	0
DHA	213	38	250	63–250
Total LC n-3 PUFA	267	47	314	79–314
**Meat, fish, eggs, enriched foods and fish oil supplements (n 94)**				
EPA	87	6	75	5-321
DPA	86	6	76	4-422
DHA	128	11	96	8-632
Total LC n-3 PUFA	302	20	268	17-1145

**^1 ^**LC n-3 PUFA - Long Chain Omega-3 Polyunsaturated Fatty Acid; SEM - Standard Error of the Mean; EPA - eicosapentaenoic acid; DPA - docosapentaenoic acid; DHA - docosahexaenoic acid

### 3.4. Differences in Food Intakes for Women ‘at risk’ and ‘not at risk’ of Developing post Natal Depression

There were no differences in the LC n-3 PUFA intakes for women ‘at risk’ and ‘not at risk’ of developing PND. There were no differences in grams per day for intakes for eggs, fish/seafood, meat and enriched food consumption between women ‘at risk’ and women ‘not at risk’ of PND (results not shown). There were no significant differences in intakes for EPA, DPA, DHA or total LC n-3 PUFA between the ‘at risk’ and the ‘not at risk’ groups. Even though this table suggests ‘at risk’ women had 20% higher DHA intakes than ‘not at risk’ women, this is due to one outlier in each of the two groups. These outliers consumed 588mg DHA (from ‘not at risk’ group) and 632mg DHA (from ‘at risk’ group). When these two outliers were deleted from the analysis, the mean DHA intakes in the ‘not at risk’ group and the ‘at risk’ group became 119mg and 118mg, respectively. The differences in LC n-3 PUFA intakes for women ‘at risk’ and ‘not at risk’ of developing postnatal depression is shown in Table 4.

**Table 4:** Comparison of LC n-3 PUFA intakes between pregnant women ‘not at risk’ and ‘at risk’ of developing PND as assessed by the Edinburgh Postnatal Depression Scale **^1^**.

Fatty acid	Women ‘not at risk’ of developing PND (n 76)			Women ‘at risk’ of developing PND (n 18)			P value^*^
	Mean	SEM	Median	Mean	SEM	Median	
EPA mg/day	87	7	78	91	14	70	0.90
DPA mg/day	84	7	77	94	12	76	0.55
DHA mg/day	123	11	96	150	37	106	0.92
LC n-3 PUFA mg/day	294	21	270	344	60	253	0.77

**^1^** PND - Postnatal Depression; SEM - Standard Error of the Mean; EPA - Eicosapentaenoic acid; DPA–docosapentaenoic acid; DHA - docosahexaenoic acid; LC n-3 PUFA - Long Chain Omega-3 Polyunsaturated Fatty Acid ^*^ P < 0.05 (Wilcoxon Nonparametric test)

There were no differences in EPA, DHA and LC n-3 PUFA intakes as assessed by the LC n-3 PUFA questionnaire between pregnant and non-pregnant women, however DPA intakes were significantly higher in pregnant women (Table 2). Oily fleshed fish and seafood generally, particularly when harvested from cold-water regions, are a rich source of EPA and DHA [35], whereas meat products, such as beef and lamb, contain a much higher DPA content [1]. As the pregnant women consumed more meat than the non-pregnant women this would explain the differences in DPA intake between the two groups. In addition 5 of the 33 non-pregnant women in the study were vegetarians, which would contribute to the lower DPA intakes, whereas there were no vegetarians in the pregnant women group. However, the majority of the vegetarians (4 out of 5) still ate fish and eggs and a further analysis excluding the vegetarians still resulted in lower DPA intakes in the non-pregnant group (data not shown). Nevertheless, the overall intake of total LC n-3 PUFA was not significantly different between the pregnant and non-pregnant women groups. 

The non-pregnant women group included eight women up to 14 years older than the pregnant women, therefore an analysis excluding all women aged > 41, was conducted. These results were the same as those found including the older women (data not shown). Furthermore, estimates of the LC n-3 PUFA dietary intakes in both the pregnant and non-pregnant women compared favourably with the results of the NNS95 (Table 2). However, given that LC n-3 PUFA consumption is skewed to the right, comparisons of median intakes from both the pregnant and non-pregnant groups to the mean intakes in the NNS95 were not significantly different. Hence our results indicate that pregnant women in this study do not necessarily consume a higher intake of LC n-3 PUFA compared to non-pregnant women.

To our knowledge there are no other studies that have compared individual LC n-3 PUFA intake between pregnant and non-pregnant women. However, two previous longitudinal Dutch studies that reported the total n-3 PUFA (including ALA) of women during pregnancy found no significant changes in their dietary intakes pre and post-pregnancy [36,37]. The Otto *et al.* [36] study measured the amount of n-3 PUFA from a group of women pre-conception and then later at 10 weeks of pregnancy. While the Al *et al.* [37] study (1996) measured intakes in women within the first 13 weeks, at 22 weeks and again at 32 weeks of pregnancy and found no differences in LC n-3 PUFA intakes.

Our results also show that there were no differences between the amount of daily LC n-3 PUFA consumed between women ‘at risk’ and ‘not at risk’ of developing PND groups (Table 4). To date there are no other studies that have used a validated FFQ to compare the LC n-3 PUFA intakes from foods such as meat, seafood, eggs, n-3 PUFA enriched foods and fish oil supplements in women ‘at risk’ and ‘not at risk’ of developing PND. Even so these findings are consistent with a previous study between non-pregnant women aged 23–97 years categorised as depressed or non-depressed [38] where they found no difference in LC n-3 PUFA intakes. However, one limitation of this study is the limited number of women at risk of developing PND available in the study. It is likely that women with depression may have been less likely to participate in a study or that some of the women screened as ‘not at risk of developing depression’ at the time of the study recruitment, later developed depression [33]. Unfortunately, data on the women who later developed postnatal depression was not collected, as a second EPDS is not routinely administered, at the hospital, to women after they have given birth. Therefore it is important for hospital staff to screen all pregnant women during an antenatal visit to identify possible cases that maybe ‘at risk’ of developing PND. A lower EPDS cut-off score (≥10) was used by the hospital to identify all possible cases of depression. Using a cut-off score ≥10 as opposed to ≥13 or ≥15 will increase the sensitivity of the EPDS to reduce the number of false negatives but may increase the number of false positives [31]. A validation study of the EPDS during pregnancy reported that a 14/15 cut-off score correctly identified all women with major depression (sensitivity 100%). Whereas using a lower 12/13 cut-off score identified the women with major depression and minor depression but also included some false positives [32]. The hospital, in this study, intentionally used a lower EPDS cut-off score (≥10) as a safety net to ensure that no women with antenatal depression would be missed. Therefore, women who scores 10 or greater on the EPDS were regarded as ‘at risk’ of developing PND and were referred to a local community centre for counselling. Changing the cut-off scores to ≥13 and ≥15 to minimize false positives still did not produce any differences in LC n-3 PUFA intakes between the groups ‘at risk’ or ‘not at risk’ of developing PND (results not shown). 

In this sample, the % rate of women ‘at risk’ of developing PND, using an EPDS cut-off score of ≥10, ≥13 and ≥15 were 19%, 13% and 12%, respectively, with a mean EPDS score 6.2 (SD 5.2). In comparison the Australian *beyondblue* Postnatal Depression program [39] where 40,333 pregnant women were screened for depression using the same EPDS cut-off scores was 20%, 9% and 5%, respectively, with a mean EPDS score 6.6 (SD 4.4). However, the pregnant women from our study deemed ‘at risk’ was screened at an average of 20 weeks gestation compared to the *beyondblue* PND program where women were screened at an average of 26 weeks gestation [39]. However, 52% of women in the *beyondblue* PND program were recruited from the state of New South Wales. They were screened at an average of 16 weeks and % rates of antenatal depression, using a EPDS cut-off score of ≥10, ≥13 and ≥15, were 17%, 6% and 4%, respectively, mean score 7.5 (SD 3.9) [39]. Our results show that our pregnant women screened for antenatal depression, had a similar prevalence rate to the *beyondblue* PND program when using the EPDS cut-off score of ≥10 [39]. Therefore our small study sample is a representative sample as the EPDS cut-off score of **>**10 was used.

For this study, a difference of 200mg/day of LC n-3 PUFA was used in a power calculation to detect a significant difference between the women ‘at risk’ and ‘not at risk’ of developing PND. Therefore a sample size of 75 women was required for each group. While this was achieved for the ‘not at risk’ of developing PND group (n = 76) the ‘at risk’ of developing PND group contained only 18 women. A further power calculation for DHA alone (with a mean of 200mg/day and a standard deviation of 95 as found in our data results) showed that if the ‘at risk’ of developing PND group did contain 75 women the intakes would still not be significant. To detect a significant difference the sample size required would require at least 710 women total. Furthermore, Table 4 suggests that intakes of DHA in women ‘at risk’ of developing PND is higher than women ‘not at risk’ of developing PND. Therefore if this study had the statistical power, the results would not support our hypothesis.

Whether or not a difference of 200mg/day of DHA would have any clinical affect between women ‘at risk’ for depression or ‘not at risk’ for depression is still unclear. For this study, although not significantly different, the ‘not at risk’ women consumed 10 mg less DHA (96mg DHA) compared with the ‘at risk’ women who consumed 106mg DHA (Table 4). Whereas, Koletzko *et al.* [24] and The International Society for the Study of Fatty Acids and Lipids (ISSFAL) [40,41] recommend during pregnancy at least 200mg/day of DHA alone, the median DHA intake found in our study and the NNS95 (83 mg/day) are much lower than this.

If DHA intakes are no different in women ‘at risk’ versus ‘not at risk’ of developing PND, then other factors may be considered. This includes metabolic abnormality in lipid metabolism or oxidative damage caused by smoking [42]. Furthermore, social and economic factors may also contribute to the risk of developing depression [43]. 

There are various recommendations for consumption of LC n-3 PUFA and they range from 135 to 1600mg [20]. In Australia the National Health and Medical Research Council (NHMRC) Nutrient Reference Values, and recommend an adequate intake (AI) for LC n-3 PUFA at 160mg/day for adult men and 90mg/day for adult women [44] but these recommendations are based on median/mean intakes of the Australian population [1,20]. In pregnancy these AI recommendations are higher at 110mg/day for 14-18 year olds and 115mg/day for 19-50 year olds [44]. The NHMRC also have Suggested Dietary Target (SDT) intakes for optimal health and the SDT for LC n-3 PUFA is 610mg/day for men and 430mg/day for women [44] and these are based on 90**^th^** percentile of Australian population intakes [1,20]. Whilst it is difficult to establish an exact amount of DHA needed to supply the needs of the growing and developing foetus and replenish the mother’s maternal stores [10], there are recommendations specifically for DHA. The ISSFAL proposed an adequate intake for pregnant women to consume > 200mg/day [40,41] and the European consensus statement proposed at least 200mg/day of DHA alone [24].

In this study the median intake shows that 50% of the women consumed an LC n-3 PUFA and DHA intake less than 268mg/day and 96mg/day, respectively. Furthermore, only 9% of the women consumed the proposed ideal recommended intake of > 200mg/day of DHA during pregnancy and lactation [40,41]. If the foetus accumulates on average » 67 mg DHA/day from the maternal supply during the last trimester of pregnancy [45], as predicted from post-mortem studies [46], then the mother will have very little or no DHA left over to provide for her own physiological needs [10]. Maternal plasma concentrations of a range of fatty acids are increased during pregnancy [12,47] among these are the LC n-3 PUFA. There is no evidence of increased maternal LC n-3 PUFA intake during pregnancy [36,37] so it is likely that the majority of these LC n-3 PUFA are mobilised from maternal stores with some contribution from oestrogen stimulated conversion of ALA to DHA [18]. Interestingly the growing foetus preferentially takes up DHA and the omega-6 PUFA called arachidonic acid (AA, 20:4n-6) and these two fatty acids become important building blocks for the foetal brain [12]. There is some evidence that women during pregnancy become deficient in DHA and as pregnancy continues their DHA deficiency index (as defined by the 22:5n-6/22:4n-6 ratio) increases during pregnancy [12]. This DHA deficiency returns to normal 6 months post-partum [47]. However this is not observed in all populations [47]. It should be noted that it is difficult to deduce net transfer of fatty acids from mother to foetus from random measurement of maternal steady state plasma concentrations. Nonetheless, the high foetal demand and absolute requirement for DHA would suggest that pregnant women are under stress in terms of providing enough DHA to her growing foetus. A recent study reported that pregnant women lack the knowledge of the importance of LC n-3 PUFA consumption during pregnancy [48]. Therefore, there is obviously a need to educate women of childbearing age about the benefits of increasing their LC n-3 PUFA intakes [48] and the best alternatives are by consuming more fish products [20].

There are various reports on actual intakes for women of reproductive age as outlined by Sioen *et al.* [49]. The actual intakes of EPA range from 46 to 278mg/day, DPA intakes range from 10 to 75mg/day, DHA intakes range from 67 to 497mg/day and LC n-3 PUFA intakes range from 156–775mg/day [49]. For our study, both pregnant and non-pregnant women, the average total LC n-3 PUFA intake of 263mg/day and 241mg/day, respectively, meets the AI recommendations of the NHMRC but falls short of the NHMRC SDT recommendations and the recently released National Heart Foundation of Australia’s recommended EPA and DHA intake of 500mg/day for healthy adults including pregnant women [50] as well as the recommendations by ISSFAL [40,41] and the European consensus [24].

The results of our study showed no difference between the average amount of seafood, meat, eggs and enriched foods (grams per day) consumed between pregnant women ‘at risk’ of developing PND and those ‘not at risk’ of developing PND. In contrast to our study, a cross-sectional national New Zealand study of 4644 adult men and women (15+ years) reported that non-fish eaters scored significantly lower on a mental health test (poorer mental health) compared to the fish eaters’ on the same mental health test (better mental health) [51]. Unlike our study, fish consumption was categorised into those who consumed no fish of any kind and those who consumed some kind of fish (ranging from ≥1 fish serving per month to ≥ 2 per day) [51]. However, the National New Zealand study did not include and compare other foods that contain LC n-3 PUFA such as meat or enriched foods.

From the current study, 51% of the pregnant women were supplementing their diet with LC n-3 PUFA enriched foods such as milk, bread, eggs and fat spreads and/or fish oil supplements (Table 3). This contributed to a total of 12% of their daily LC n-3 PUFA intakes; 5% EPA, 1% DPA, 22% DHA. Enriched foods can be very useful to top-up daily levels of LC n-3 PUFA when other enriched foods like fish and meat have already been incorporated into the diet [52]. A recent study [53] found that a group of overweight subjects with a habitual low fish intake could significantly increase their daily LC n-3 PUFA intake from 258mg to 1195 mg/day by incorporating up to eight various LC n-3 PUFA enriched foods into their diet. This same study showed that consumption of these enriched foods resulted in increased erythrocyte LC n-3 PUFA levels to a similar extent than if the subjects consumed fish/seafood [54].

A limitation of this study was the lack of blood biomarkers from the women, such as erythrocyte LC n-3 PUFA levels which are indicative of tissue LC n-3 PUFA levels. However, a previous study found using the same FFQ that 53 healthy adults reported LC n-3 PUFA intakes parallel levels analysed in erythrocytes and plasma and these intakes [27,28] were also comparable to those reported in the NNS95. Another limitation of our current study is the comparability of the pregnant and non-pregnant women. Pregnant women were recruited from a hospital, whilst the non-pregnant women were recruited from the University of Wollongong and the wider Wollongong community. Whilst it is recognised that women with a higher level of education or income were more aware of issues related to LC n-3 PUFA [48], however, the results of our current study compare favourably to the intakes of women assessed in the NNS95, which covers a wide range of education and income levels. It must also be noted there was a difference in methodologies used between this study (FFQ including enriched foods) and the NNS95 (24 hr food recall and FFQ) [30]. As well a difference of 9 years between this study (data collected 2004) and the NNS95 and within that time frame the availability of LC n-3 PUFA foods and the dietary habits of individuals have changed, especially with the introduction of LC n-3 PUFA enriched foods. For that reason we compared the LC n-3 PUFA intakes of pregnant and non-pregnant women, excluding the enriched foods, and their intakes compared favourably to those intakes of women reported in the NNS95. Furthermore due to limited time for subject recruitment 75 women for each of the groups was not achieved as only 18 women ‘at risk’ of developing PND and 33 non-pregnant women, compared to 76 women ‘not at risk’ of developing PND were recruited for this study. However, the data were also compared to that of the NNS95 which contained 5770 women.

In summary the LC n-3 PUFA intakes did not differ between pregnant women and non-pregnant women and were comparable to the intakes as recorded by the Australian NNS95. In our study, the average total LC n-3 PUFA intake for both pregnant and non-pregnant women meet the recommendations of some but not all recommended ranges for healthy adults. The LC n-3 PUFA intakes did not differ between the pregnant women ‘at risk’ of developing PND and ‘not at risk’ of developing PND. Although there is an impression that inadequate LC n-3 PUFA intakes in pregnant women may predispose them to developing postnatal depression due to growing foetal organ systems (particularly the nervous systems) requiring increased amounts of LC n-3 PUFA [23], the results of this study do not currently support this deficiency concept.

## 4. Conclusion

Dietary LC n-3 PUFA intakes did not differ between pregnant and non-pregnant women nor did they differ between women ‘at risk’ and ‘not at risk’ of developing PND. Furthermore, the average total LC n-3 PUFA intakes for both pregnant and non-pregnant women are low compared to recommendations from various organisations.

## Acknowledgments

The authors would like to acknowledge the University of Wollongong for funding the study, the hospital staff for their assistance and the study volunteers for their participation in the study and Pam Davy, and Ken Russell, for their assistance with statistical analysis.
